# The application of Item Response Theory on a teaching strategy profile questionnaire

**DOI:** 10.1186/1472-6920-10-14

**Published:** 2010-02-10

**Authors:** Ulf Brodin, Uno Fors, Klara B Laksov

**Affiliations:** 1 Department of Learning, Informatics, Management and Ethics (LIME), Berzelius väg 3 Karolinska Institutet, 171 77 Stockholm, Sweden

## Abstract

**Background:**

In medical education research, various questionnaires are often used to study possible relationships between strategies and approaches to teaching and learning and the outcome of these. However, judging the applicability of such questionnaires or the interpretation of the results is not trivial.

**Methods:**

As a way to develop teacher thinking, teaching strategy profiles were calculated for teachers in a research intensive department at Karolinska Institutet. This study compares the sum score, that was inherent in the questionnaire used, with an Item Response Theory (IRT) approach. Three teaching dimensions were investigated and the intended sum scores were investigated by IRT analysis.

**Results:**

Agreements as well as important differences were found. The use of the sum score seemed to agree reasonably with an IRT approach for two of the dimensions, while the third dimension could not be identified neither by a the sum score, nor by an IRT approach, as the items included showed conflicting messages.

**Conclusions:**

This study emphasizes the possibilities to gain better insight and more relevant interpretation of a questionnaire by use of IRT. A sum score approach should not be taken for granted. Its use has to be thoroughly evaluated.

## Background

Questionnaires are regularly constructed to catch underlying concepts, or variables, which can not be directly measured. An example of such a questionnaire is designed by Professor Jan Vermunt at University of Leiden, Netherlands. The aim of this questionnaire is to generate thinking about ones teaching practice by revealing tendencies in a teacher's teaching practice towards the 'taking over' or 'activating' of a) the application of knowledge, b) meaning of knowledge and c) reproduction of knowledge [1]. This questionnaire was not intended as a tool for research, but a way to provide feedback to teachers on their teaching strategies as an intervention to develop teaching practices at the department. The results of this intervention study can be found in [2].

The inventory consists of eighteen propositions on which teachers had the opportunity to grade how often they performed certain teaching-related activities on a Likert-type scale, ranging from 1-5. The propositions were linked to either an 'activating' approach, (e.g. Make students formulate their own point of view) or a 'taking over' approach (e.g. Give examples or ask detailed questions) and based on the three themes mentioned above; meaning, application and reproduction. Vermunt's inventory is based on these six aspects of teaching. According to a template teachers could summarize their scores and draw a graph, which indicated their teaching strategy profile, and which in turn was used to encourage discussion on teaching issues.

Further explanation of these constructs and related discussion can be found in [2] and [3].

In this study we concentrate on Activating Application (AA), Activating Meaning (AM) and Activating Reproduction (AR). These three sets of items should, according to Vermunt, detect to which extent he/she let the student give applied examples (AA), how the teacher makes the student show that they have understood (AM) and to which extent he/she encourage the student to repeat knowledge (AR). The sum scores for AM, AA and AR are thought to reflect three dimensions of teaching practice, that can be viewed as underlying latent traits. Although AA, AM and AR are easily described and their attributes can be listed, they can not be measured directly, since the variables are concepts rather than physical dimensions. The doubt of using raw scores for analysis is well founded and often pointed out [4].

It is a well known problem that moving a questionnaire concept from one population to another might alter the validity and other characteristics, which were found appropriate for the original population, on which the questionnaire was developed [5]. Unfortunately, the appropriateness of a sum score approach or similar is seldom thoroughly validated [5]. As we are dealing with an underlying, latent trait there is no clear answer. We have to rely on indication and plausibility. But in medical education research, there is a need for more stable and verified methods for interpreting and analysing the results of questionnaires when the aim is to sustain consequential changes that are introduced. It is important to study the applicability of research tools like questionnaires in different populations as well as possible new ways to draw conclusions from such methods.

The aim of this study is to apply an Item Response Theory (IRT) approach, and evaluate whether the sum score approach still seems valid for the study population from Karolinska Institutet. By ranking the teachers according to the two methods, a pragmatic comparison might be obtained.

## Methods

Fifty-nine (59) volunteering teachers, participating in a teacher training course at Karolinska Institutet, were asked to fill in the Vermunt questionnaire. Their responses were then scored by summarising the coded answers to the items on the questionnaire, see Additional file 1 and [3].

Three responses, each on an ordered 5- point Likert type scale, from each of the AA, AM and the AR were collected.

When defining a latent trait, i.e. an underlying unobservable variable, such as attitude or social competence, the location and variability of such a measure is arbitrary as well as it's distribution.

Different coding or headlining will change all these characteristics. However, the characteristics of the respondents in terms of ranking on a latent trait axis should be appropriate and invariant to the choice of coding.

The most straightforward approach underlying latent traits is to just summarise the answers from items as they are coded. In the questionnaire evaluated in this study (see Additional file 1), the items are coded on an ordered 5- point scale. A large sum is thought to indicate a 'high degree of activating', and a low sum a 'low degree of activating'.

An alternative approach is offered by the Item Response Theory (IRT), where the actual perception of the items can be accounted for. The ideas from IRT can be applied in many ways, see [5] for a general discussion. One such approach will be evaluated in this study and compared with the conventional sum score. The methods are briefly described below. Further information about the mathematical theory can be found in [6] and [7]. For this study, we have chosen a 2PL model with item specific discrimination. The more parsimonious 1PL model, with equal discrimination for all items, could have been chosen. However, we have to allow a flexible model so that a possible rejection of the intended raw sum score approach does not depend on an application of a too parsimonious model. Models with even more complexity (e.g. with both item and category specific discrimination) are out of the question due to too many parameters in the relation to the moderate sample size. The actual sample size might be seen as too small even for a 2PL model, but its flexibility is needed and it should be emphasized that no final model is looked for. The estimated models will, of necessity, be very approximate with large SE:s for the parameters, but can nevertheless constitute a basis for an evaluation of some basic characteristics of the questionnaires and the use of the raw sum score (or some transformation of the sum score) as a relevant measure.

### Method 1, the sum score approach

Let us assume that the location (the difficulty) is the same for all items within a dimension and that there is a constant distance between sequential categories within questions. This will correspond to the definition of the sum score approach.

The sum score might be in good agreement with the underlying degree of activating, but may also be far from the intended if the items are not suitable for being summarised. The well known main requirements for a sum score to work are the following:

1. The distances between the steps in the graded scale are constant and equal.

2. The difficulty, or the weight of the item, is the same for all items.

3. The items should work in the same direction towards a common underlying trait (often called scalability).

Even if these characteristics are intended at the construction of the questionnaire, the population, on which it will be applied, may perceive the items differently. This risk of misunderstanding is even more cumbersome when the questionnaire is 'moved' to be applied on a different population, in a different environment with, for example, a different language or culture.

The above stated characteristics of the set of items are fixed in advanced and do not take into account how they might change when applied. Under the classical test theory, the teacher's test score is the sum of the scores received on the items of the test. A teacher's latent trait is calculated according to an external fixed scale, decided independently of the intended population. The basis is usually (or should be) some reference set of individuals. However, usually there is no straightforward linear relationship between the sum score and a position on the constructed latent trait.

Thus, the answers as coded are summarised like this:

SumAA = Q4+Q7+Q17;, SumAM = Q6+Q10+Q15; and SumAR = Q2+Q9+Q13.

SumAA, SumAM and SumAR represent the tendency of teachers to more or less activate the students' activities that have been suggested important for learning. This 'latent trait' is hereafter called 'teacher tendency' with respect to Activating Application, Activating Meaning and Activating Reproduction.

### The problem of non response

When, for some respondents, there is a 'non response' to a particular question, this has to be taken care of in order to create justified abilities for all respondents. In other words, values have to be imputed. A simple and reasonable method is to look for colleagues with similar profiles as the participant with a non response. The median or most frequent value in this set will then be imputed for the non response. Such a procedure can be refined by an iteration procedure, but this will not be done in this case as there are rather few 'missing data'. A disadvantage of this method is a bias towards a more homogenous sample (i.e. a more favourable sample) than could be expected from a complete sample. A more complicated situation arises when the non responses are not due to missing but rather that the question is interpreted as irrelevant for the respondent (something we seldom know). In such a case, no value should be imputed.

In this material, there is just one non-response for the AA dimension. There is only one colleague with the same profile so the imputation is simple for this case.

For the AR dimension there are 7 'non responses', all from Q13. Obviously, these can not be considered as values missing at random. An imputation might be applied according to the 'simple profile' principle to get a sum score for these 7 teachers. The teacher tendency under IRT might be estimated using an IRT model, without any imputation of individual values. Unfortunately, the evaluation revealed that the sum score does not work, nor could a reasonable IRT model be found. As a consequence, the imputation/estimation was abandoned and the sample of 52 complete teacher responses was used.

The AM dimension is complete.

### Method 2, the IRT approach

Under Item Response Theory, the primary interest is the teacher's score on each individual item, rather than on the test sum score.

### The parametric IRT approach

As for the sum score approach, a latent scale is constructed or identified. However, the advantage of IRT is it's independence of the coding. An individual's position on the scale is estimated from the answer profile. This profile is related to the difficulty of the item and its item thresholds (characterised by the over all relative frequency of answers to the different item levels) as well as the item's quality, which in essence means the item's ability to discriminate between individuals on the scale. The item difficulty is anchored at a location on the latent trait.

The individual values on the latent trait scale are directly related to the odds of answering at different levels of the items. The higher the score, the larger the probability to answer on high levels in a positively ordered item set. To allow flexibility without too many parameter estimates, the so called 2PL graded response model is chosen.

For further details of the actual IRT approach, see Additional file 2.

### The nonparametric 'Mokken scalability' approach

The Mokken scalability analysis [6] is an efficient method in evaluating to which extent (scalability) the items in a questionnaire work together to form one underlying latent trait. However, it does not estimate the teacher tendency, just evaluate whether the respondents can be reasonably ordered by the sum score. Three measures are essential in such an analysis:

1. The item pair scalability, H_ij_, in essence the correlation between two ordered variables.

2. The item scalability, H_i_, an item's correlation with the remaining set of variables.

3. The item set scalability, H, the total correlation for the set of variables.

The scalability can be viewed as the observed correlation divided by the maximum correlation for the observed data (which, in contrast to continuous variables, is < 1.)

An over all requirement is that the scalabilities according to 2 and 3 above must be positive.

If a reasonable scalability is found (a rule of thumb is > 0.3), the sum score approach might be accepted.

The characteristics of the scoring procedure should, ideally, be calculated from a large reference set, and then applied on the actual 'test set'. However, as is often the case, no reference set is available, which leads us to use the actual sample as its own reference.

In this study, the questionnaire will be evaluated in two steps:

1. A nonparametric scalability analysis. If the scalability is found not sufficient, the sum score can be rolled out as inappropriate.

2. A parametric IRT model will be applied to investigate item difficulty and item discrimination.

The assumption of three latent traits, AA, AM and AR, implies that three separate analyses are needed for this study.

The scalability analysis is performed by the Mokken program [8].

The parametric IRT approach is evaluated by the Parscale computer program [9].

## Results

### The sum score approach

The item responses, with imputed values, were used in the calculations. There are no strong correlations (in terms of squared correlation coefficients) between the three sum score dimensions AA, AM and AR. A certain relationship is seen between AA and AM, r^2 ^= 0.34, while r^2^(AA, AR) and r^2^(AM, AR) < = 0.01. These low relationships indicate that a total sum score, AA+AM+AR, suffers from lack of a common latent trait.

A simple measure of the reliability of one underlying dimension is the Cronbach's coefficient alpha of the internal consistency. It is commonly accepted that an α > 0.7 indicates a good reliability that the items are measuring the same underlying construct. In this study the alpha values were as follows:

α_AA _= 0.63, α_AM _= 0.54, α_AR _= 0.07. Thus, α_AA _and α_AM _can be barely accepted but α_AR _indicates conflicting items within AR.

### The IRT approach

#### The Mokken nonparametric scale analysis

The result of the Mokken analysis is shown in Table 1. The low item scalabilities indicate weak scales, saying that sum scores from AA and AM might be used to separate teachers which are ranked a considerable distance from each other while the ranking of teachers with nearby scores might be mixed up. The analysis of AR indicates that the sum score is not a suitable measure for ranking teachers in this population. The scalability for the total set of items (H = 0.122) is clearly not sufficient for a reliable simple sum score.

**Table 1:** Scalability estimates

Trait	Item scalability*			Item set scalability**
Activating	Q_4_	Q_7_	Q_17_	
Application	0.322	0.390	0.447	0.387

Activating	Q_6_	Q_10_	Q_15_	
Meaning	0.270	0.374	0.354	0.331

Activating	Q_2_	Q_9_	Q_13_	
Reproduction (n = 52)	0.0285	-0.1659	0.0486	-0.0264

Total AA+AM+AR				0.122

*The item's capability to work together with the other 2 items in the set.

** The capability of all 3 items to work together.

### The parametric IRT approach

The result for Activating Application, AA, is found in Table 2.

**Table 2:** Estimated AA - parameters and their SE.

Item	Discrimination (SE)	Location (SE)	p-value for item	
Q4	0.944 (0.214)	-0.028 (0.223)	0.180	

Q7	0.867 (0.183)	0.727 (0.229)	0.738	

Q17	0.888 (0.178)	0.771 (0.209)	0.179	

Total			0.265	

Thresholds related to the location	c_1_	c_2_	c_3_	c_4_
	
	1.438	0.486	-0.422	-1.501

Distance between thresholds	-	0.952	0.908	1.079

Prob. from χ^2 ^- fit statistic

The discriminating power is about the same for the items within AA and not far from 1.0, which can be considered adequate as viewed from a Rasch perspective (7).

**Figure 1:**
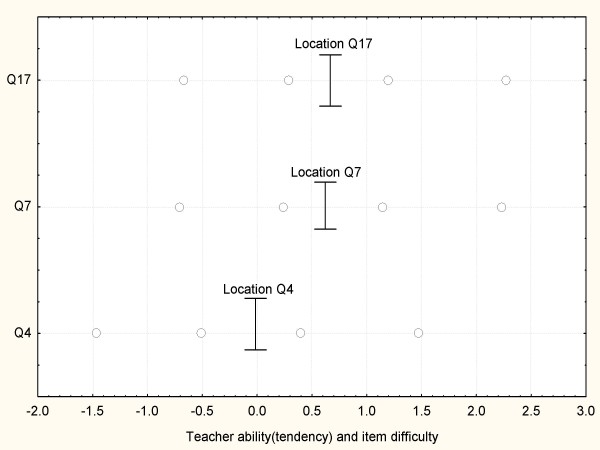
**Item thresholds at the latent trait**. o = category thresholds.

**Figure 2:**
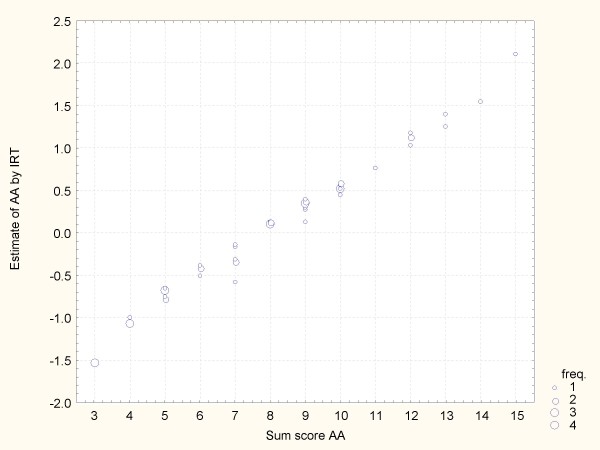
**IRT estimates of teacher AA vs the Sum score**.

Q4 is considered an easier item to endorse than is Q7 or Q17 as indicated by the value -0.028. A rough test, based on normality and independence assumption (using location and SE estimates from table 2), yields a z > 2.3 for Q4 against Q7 and Q17, which indicates a systematic difference (p < 0.05) between the locations. Q7 and Q17 are anchored at the same position, meaning that they in essence reflect the same part of the latent trait. The message is that one of the questions should be reformulated to get further information from the respondent. The relationship between item difficulty (location) and the thresholds are illustrated in fig. 1. There is a good agreement between the IRT estimated tendency and the sum score, see fig. 2. However, a few teachers with equal sum scores are separated by the IRT approach.

A χ^2^-statistic = 0.265 does not indicate any systematic violation of the chosen IRT model.

The result for Activating Meaning, AM is shown in Table 3.

**Table 3:** Estimated AM - parameters and their SE.

Item	Discrimination (SE)	Location (SE)	p-value for item	
Q6	0.837 (0.164)	0.253 (0.233)	0.246	

Q10	0.542 (0.155)	2.547 (0.429)	0.296	

Q15	0.738 (0.133)	0.067 (0.249)	0.321	

Total			0.231	

Thresholds related to the location	c_1_	c_2_	c_3_	c_4_
	
	1.774	0.576	-0.470	-1.879

Distance between thresholds		1.198	1.046	1.410

Prob. from χ^2 ^- fit statistic

The discriminating power is somewhat lower than for AA but the items seem to work reasonably. Item Q6 and Q15 turn out to be fairly easy to endorse while Q10 is a 'difficult' item. A rough test of Q10 (as done for AA) yields a systematic difference (p < 0.01) against Q6 and Q15, z > 3.0.

**Figure 3:**
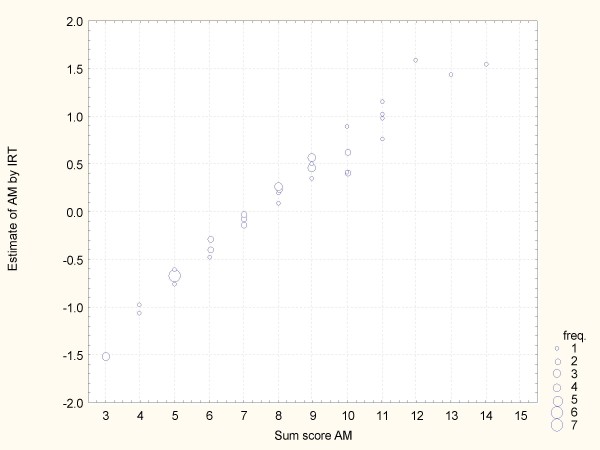
**IRT estimates of teacher AM vs the Sum score**.

There is a reasonable agreement between the IRT estimated tendency and the sum score, see fig. 3. However, the IRT approach change the ranking for a few teachers, particularly for those with a sum score ≥ 10

The χ^2^-statistic = 0.231 does not indicate any systematic violation of the chosen IRT model.

The result for Activating Reproduction, AR, is shown in Table 4.

**Table 4:** Estimated AR - parameters, and their SE, n = 52.

Item	Discrimination (SE)	Location (SE)	p-value for item	
Q2	0.206 (0.033)	-1.077 (0.812)	0.575	

Q9	0.258 (0.041)	-0.339 (0.614)	0.427	

Q13	0.142 (0.033	-2.990 (1.075)	0.111	

Total			0.326	

Thresholds related to the location	c_1_	c_2_	c_3_	c_4_
	
	4.322	1.378	-1.353	-4.348

Distance between thresholds		2.944	2.731	2.995

Prob. from χ^2 ^- fit statistic

Estimated parameters are shown based on respondents with complete questionnaires (n = 52). The estimated discriminating power is very low for these items and the estimated locations have a large SE, which is in agreement with the very low Cronbach's α.

**Figure 4:**
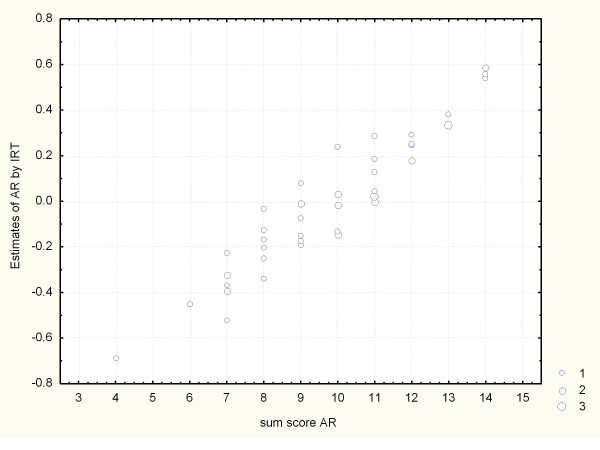
**IRT estimates of teacher AR vs the Sum score**.

The result guides us to mistrust the items in constituting an underlying latent trait. There is only a weak agreement with the sum score, see fig. 4.

Also here, the χ^2^-statistic = 0.326 does not indicate any systematic violation of the chosen IRT model. This does not mean that the model is adequate, but rather that it is difficult to find any model for these heterogeneous data. A more simple model is probably sufficient but the same model is kept in order to view AA, AM and AR simultaneously.

### Comparison between the sum score and the IRT approach

Fig. 2, 3 and 4 can be used for comparison of the rankings. With a straight line, drawn between any two points, a negative slope identifies a reversed ranking by the two methods. For the AA and AM dimension there is a reasonable agreement. The thresholds are approximately equal to one, as is assumed by the sum score approach. The main disparity is the difference in item difficulty (location). For AA item Q4 is considered more easy (-0.028) than are Q7 and Q17 (about 0.7), while the sum score approach assumes equality. Just a few ranks are shifted, respondents with a sum score of 6 or 7.

For AM, Q10 is considered much harder than are Q6 and Q15. This is the main cause for the shifted ranks for respondents with a sum score > 9.

For AR, the two different methods do not agree very well. The interpretation is that none of the methods can sufficiently identify an underlying latent trait.

## Discussion

The sum score, based on the Vermunt questionnaire, is thought to represent the AA, AM and AR dimensions and thereby estimate, or rank, the teachers. The low correlations between AA, AM and AR, together with the scalability analysis, indicate that we are really measuring three separate latent traits.

The interpretation of the questionnaire might change when it is moved and presented to another population. If the sample of medical teachers could be considered as a sample from the population underlying the construction of the Vermunt questionnaire, we would expect a reasonable agreement between the sum score approach and a suitable IRT approach. At least, it should be approved by the Mokken scale analysis.

Although the material is limited, just 59 medical teachers, the IRT approach indicates that characterising the teachers by a sum score might not be the best, or not even a good choice.

It might be argued that this material (59 teachers) is far too small for estimation of IRT models. This is certainly true, but the modelling should be considered rather as indicative and a guidance in viewing the use of raw sum scores. Inevitably, small questionnaire studies are carried out and must not be ignored with respect to analysis and preliminary validation. They should be thoroughly analysed in order to find, at an early stage, changes and possible improvements for further application.

Both the sum score and the IRT tendency produce artificial latent scales. The sum score has no direct correspondence to the answer profile and differences between sum scores are not clearly related to differences in profiles. However, the IRT tendency directly relates to the probability of a specified answer to a specified question and differences between abilities can be directly transformed to differences in probability for different answer profiles. Furthermore, IRT creates item specific weights, which yield a possibility to discriminate teachers with equal sum scores but different profiles. These differences are usually quite small and unimportant when we take the SE into account. There are, however, some interesting results. Looking at AM, it could be noticed that the IRT approach like to rank the teachers somewhat differently, particularly those with a sum score 9 to11.

Considering AR the analysis reveals, together with Cronbach's α, that the 3 items do not co-operate in forming a latent trait. In fact, the negative Mokken scalability indicates that they are contradictory. Something has happened, - the population structure might not be similar to the reference population underlying the elaboration of the questionnaire, - the environment is a different one and the items are probably not perceived in the same way. Much can happen when we 'move' questionnaires between populations, countries and cultures. No sharp conclusions can be drawn from this limited sample, but the analysis would serve as a warning. So, our data indicates that we should not take sum scores for granted in medical education research, even if it has been proven to work in earlier studies on a different population.

It should be mentioned that the imputation of missing values in the AR set is 'a mission impossible' as no plausible common latent trait could be found. Estimating teacher tendency by the IRT is probably a better approach when we like to include incomplete cases, but no firm conclusion can be drawn in this case.

Neither the sum score, nor the IRT approach is likely to rank the teachers in a reliable way on the AR dimension.

## Conclusions

This study indicates that an IRT approach can give an insight beyond the scope of a sum score when estimating a teacher strategy profile from a questionnaire. In particular, the IRT approach is able to reveal that questions might not have equal impact when creating the latent trait, identifying 'good' and 'bad' items as well as pointing out items which do not seem to work together.

Further material, in terms of medical teachers, should be collected to investigate the degree of difference between the sum score and the IRT approach when estimating AA, AM and AR levels for medical teachers, and particularly, if the questionnaire is to be used for ranking the teachers. However, while the sum score for AA and AM seems to work reasonably, it can be concluded that the sum score for AR does not work as intended. There is a strong indication that the sum score is an unsuitable measure for the AR dimension.

This means, on the whole, the Vermunt questionnaire might not be easily transferred when moved to a population like the teachers at Karolinska Institutet. The set of items have to be enlarged to reasonably capture the AA and the AM dimension. The AR items have to be reformulated and the set enlarged.

## Abbreviations

IRT: Item Response Theory; AA: Activating Application; AM: Activating Meaning; AR: Activating Reproduction; α: Cronbach's coefficient alpha; SE: Standard Error.

## Competing interests

The authors declare that they have no competing interests.

## Authors' contributions

UB is responsible for the statistical evaluation of the questionnaire. The frequent use of questionnaires in medical studies and the routine use of sum score is the background to the special interest in more flexible and informative methods offered by IRT. KBL is responsible for conducting and accomplishment of the questionnaire study on the 59 medical teachers. UF is responsible for the introduction and parts of the background considerations. All authors read and approved the final manuscript.

## Pre-publication history

The pre-publication history for this paper can be accessed here:

http://www.biomedcentral.com/1472-6920/10/14/prepub

## Supplementary Material

Additional file 1 **The questionnaire**. The actual part of the questionnaire, the text for the item levels and the complete answer data file. Click here for file

Additional file 2 **The parametric IRT approach**. A description of the 2PL model and brief explanations of the interpretation of the parameters. Click here for file
