# Supplementary material for: The application of Item Response Theory on a teaching strategy profile questionnaire
Source: BMC Med Educ. 2010 Feb 10;10:14. doi: 10.1186/1472-6920-10-14 (PMC2830224; doi:10.1186/1472-6920-10-14)
Supplement: Additional file 1 — The questionnaire. The actual part of the questionnaire, the text for the item levels and the complete answer data file. [file 1472-6920-10-14-S1.DOC]

**Additional file 1**

# *The questionnaire*

This questionnaire is about activities that teachers undertake when they teach. Please read every statement carefully and then indicate the degree to which you use the stated activity in your teaching(circle the number). The numbers behind the statements have the following meaning:

( © Jan D. Vermunt, ICON – Graduater School of Education, Leiden University, Netherlands)

1= I do this seldom or never, 2= I do this sometimes 3= I do this regularly

4= I do this often 5= I do this almost always

Extract from the questionnaire:

Question nr Question nr

Swedish original

version version The statement Answers

… 1,2,3,4,5

Q2 Q31 Tell students exactly what they have to do

Q4 Q33 Ask about the relevance of the subject matter

for real life

Q6 Q35 Make students formulate their own point of view

Q7 Q36 Let students make connections with their own

experiences

Q9 Q38 Ask detailed questions

Q10 Q39 Give student’s assignment of making a diagram

of the subject matter

Q13 Q42 Give exams that test factual knowledge

Q15 Q44 Ask for similarities and differences between concepts

Q17 Q46 Let students solve real life problems

The scores are then summarized as indicated in the text.

Response matrix from the 59 medical teachers.

Underlined bold figure for ID 59 indicate imputed values

|  |  |  | Dimension AA | | | Dimension AM | | | Dimension AR | | | Sum | Sum | Sum |  |
| --- | --- | --- | --- | --- | --- | --- | --- | --- | --- | --- | --- | --- | --- | --- | --- |
| ID | Age | Gender | Q4 | Q7 | Q17 | Q6 | Q10 | Q15 | Q2 | Q9 | Q13 | AA | AM | AR | Total |
| 12 | 53 | M | 5 | 1 | 3 | 1 | 1 | 3 | 2 | 3 | 2 | 9 | 5 | 7 | 21 |
| 14 | 32 | M | 4 | 2 | 3 | 3 | 1 | 3 | 5 | 5 | 4 | 9 | 7 | 14 | 30 |
| 15 | 33 | F | 1 | 5 | 1 | 2 | 2 | 3 | 1 | 2 |  | 7 | 7 | 4 | 18 |
| 16 | 30 | M | 2 | 5 | 3 | 5 | 3 | 3 | 4 | 4 | 4 | 10 | 11 | 12 | 33 |
| 17 | 25 | F | 4 | 3 | 3 | 3 | 3 | 4 | 2 | 3 | 2 | 10 | 10 | 7 | 27 |
| 18 | 63 | M | 2 | 1 | 1 | 1 | 2 | 2 | 3 | 2 | 2 | 4 | 5 | 7 | 16 |
| 20 | 34 | F | 4 | 3 | 3 | 3 | 2 | 4 | 4 | 3 | 3 | 10 | 9 | 10 | 29 |
| 24 | 25 | F | 2 | 4 | 1 | 4 | 1 | 4 | 5 | 4 | 2 | 7 | 9 | 11 | 27 |
| 27 | 26 | F | 1 | 1 | 1 | 2 | 1 | 2 | 2 | 5 | 5 | 3 | 5 | 12 | 20 |
| 28 | 32 | M | 2 | 1 | 1 | 2 | 1 | 4 | 1 | 4 | 5 | 4 | 7 | 10 | 21 |
| 29 | 27 | F | 2 | 1 | 2 | 3 | 2 | 3 | 5 | 4 | 5 | 5 | 8 | 14 | 27 |
| 31 | 60 | M | 3 | 1 | 2 | 2 | 1 | 2 | 4 | 3 | 3 | 6 | 5 | 10 | 21 |
| 36 | 42 | M | 2 | 5 | 4 | 4 | 1 | 2 | 4 | 1 | 3 | 11 | 7 | 8 | 26 |
| 37 | 30 | M | 3 | 2 | 1 | 3 | 1 | 5 | 4 | 5 | 1 | 6 | 9 | 10 | 25 |
| 42 | 29 | M | 4 | 4 | 4 | 4 | 3 | 4 | 5 | 5 | 4 | 12 | 11 | 14 | 37 |
| 43 | 54 | M | 4 | 3 | 2 | 2 | 4 | 4 | 2 | 4 | 3 | 9 | 10 | 9 | 28 |
| 44 | 26 | M | 3 | 2 | 3 | 5 | 2 | 5 | 1 | 3 | 3 | 8 | 12 | 7 | 27 |
| 46 | 29 | M | 2 | 2 | 5 | 4 | 1 | 4 | 5 | 3 | 5 | 9 | 9 | 13 | 31 |
| 47 | 27 | M | 4 | 5 | 5 | 5 | 1 | 4 | 3 | 4 |  | 14 | 10 | 10 | 34 |
| 49 | 25 | F | 2 | 3 | 2 | 3 | 4 | 3 | 4 | 3 | 5 | 7 | 10 | 12 | 29 |
| 51 | 31 | F | 3 | 3 | 1 | 2 | 1 | 3 | 5 | 3 | 5 | 7 | 6 | 13 | 26 |
| 52 | 27 | M | 2 | 2 | 1 | 4 | 1 | 4 | 4 | 1 | 5 | 5 | 9 | 10 | 24 |
| 54 | 32 | F | 3 | 1 | 1 | 1 | 1 | 1 | 5 | 3 | 5 | 5 | 3 | 13 | 21 |
| 56 | 29 | F | 5 | 4 | 3 | 3 | 3 | 3 | 3 | 3 | 5 | 12 | 9 | 11 | 32 |
| 59 | 27 | F | 3 | **1** | 2 | 2 | 1 | 2 | 5 | 3 |  | 6 | 5 | 13 | 24 |
| 60 | 46 | M | 4 | 2 | 3 | 2 | 2 | 3 | 3 | 4 | 2 | 9 | 7 | 9 | 25 |
| 61 | 28 | M | 1 | 1 | 3 | 3 | 1 | 5 | 5 | 1 | 5 | 5 | 9 | 11 | 25 |
| 63 | 27 | F | 4 | 1 | 1 | 3 | 1 | 5 | 4 | 4 | 5 | 6 | 9 | 13 | 28 |
| 66 | 29 | F | 4 | 3 | 2 | 4 | 1 | 3 | 5 | 5 |  | 9 | 8 | 14 | 31 |
| 67 | 29 | F | 4 | 2 | 1 | 3 | 1 | 1 | 2 | 4 | 5 | 7 | 5 | 11 | 23 |
| 68 | 29 | F | 5 | 1 | 1 | 2 | 1 | 2 | 5 | 1 | 5 | 7 | 5 | 11 | 23 |
| 72 | 25 | F | 2 | 2 | 1 | 5 | 1 | 1 | 2 | 5 | 1 | 5 | 7 | 8 | 20 |
| 74 | 27 | F | 2 | 4 | 3 | 4 | 1 | 3 | 1 | 4 | 3 | 9 | 8 | 8 | 25 |
| 76 | 44 | M | 3 | 2 | 1 | 1 | 1 | 1 | 1 | 4 | 5 | 6 | 3 | 10 | 19 |
| 85 | 32 | M | 4 | 2 | 2 | 4 | 1 | 1 | 4 | 5 | 5 | 8 | 6 | 14 | 28 |
| 88 | 36 | F | 4 | 3 | 2 | 3 | 2 | 3 | 4 | 3 | 5 | 9 | 8 | 12 | 29 |
| 89 | 36 | M | 1 | 1 | 2 | 1 | 1 | 4 | 3 | 3 | 3 | 4 | 6 | 9 | 19 |
| 90 | 34 | F | 3 | 2 | 3 | 4 | 1 | 1 | 3 | 2 | 5 | 8 | 6 | 10 | 24 |
| 92 | 41 | F | 5 | 2 | 5 | 2 | 1 | 5 | 5 | 1 |  | 12 | 8 | 10 | 30 |
| 95 | 1 | - | 3 | 4 | 3 | 4 | 5 | 5 | 1 | 2 | 1 | 10 | 14 | 4 | 28 |
| 96 | 53 | M | 4 | 2 | 2 | 3 | 1 | 3 | 3 | 4 | 2 | 8 | 7 | 9 | 24 |
| 97 | 26 | M | 5 | 1 | 1 | 2 | 1 | 2 | 5 | 1 | 2 | 7 | 5 | 8 | 20 |
| 98 | 32 | F | 5 | 5 | 5 | 5 | 1 | 1 | 1 | 1 | 5 | 15 | 7 | 7 | 29 |
| 99 | 27 | F | 3 | 4 | 3 | 4 | 1 | 3 | 5 | 1 | 3 | 10 | 8 | 9 | 27 |

|  |  |  | Dimension AA | | | Dimension AM | | | Dimension AR | | | Sum | Sum | Sum |  |
| --- | --- | --- | --- | --- | --- | --- | --- | --- | --- | --- | --- | --- | --- | --- | --- |
| ID | Age | Gender | Q4 | Q7 | Q17 | Q6 | Q10 | Q15 | Q2 | Q9 | Q13 | AA | AM | AR | Total |
| 100 | 27 | F | 4 | 2 | 4 | 2 | 1 | 4 | 4 | 2 |  | 10 | 7 | 11 | 28 |
| 101 | 36 | M | 5 | 4 | 4 | 3 | 4 | 3 | 3 | 2 | 1 | 13 | 10 | 6 | 29 |
| 102 | 34 | M | 1 | 1 | 1 | 2 | 1 | 3 | 4 | 4 | 1 | 3 | 6 | 9 | 18 |
| 103 | 29 | M | 4 | 4 | 5 | 4 | 4 | 5 | 5 | 4 |  | 13 | 13 | 13 | 39 |
| 104 | 49 | M | 3 | 3 | 4 | 3 | 3 | 4 | 3 | 2 | 2 | 10 | 10 | 7 | 27 |
| 105 | 28 | F | 1 | 1 | 3 | 1 | 1 | 2 | 1 | 3 | 5 | 5 | 4 | 9 | 18 |
| 107 | 32 | M | 3 | 4 | 3 | 4 | 2 | 5 | 4 | 4 | 3 | 10 | 11 | 11 | 32 |
| 109 | 28 | M | 1 | 1 | 1 | 1 | 1 | 1 | 5 | 1 | 5 | 3 | 3 | 11 | 17 |
| 113 | 2 | M | 2 | 1 | 1 | 2 | 1 | 1 | 3 | 2 | 5 | 4 | 4 | 10 | 18 |
| 115 | 31 | F | 4 | 3 | 1 | 2 | 1 | 2 | 1 | 4 | 2 | 8 | 5 | 7 | 20 |
| 116 | 40 | M | 5 | 4 | 3 | 4 | 4 | 3 | 3 | 4 | 5 | 12 | 11 | 12 | 35 |
| 117 | 28 | F | 1 | 1 | 1 | 1 | 1 | 1 | 4 | 2 | 5 | 3 | 3 | 11 | 17 |
| 120 | 44 | M | 3 | 2 | 3 | 2 | 2 | 4 | 2 | 4 | 2 | 8 | 8 | 8 | 24 |
| 124 | 39 | F | 3 | 2 | 4 | 2 | 1 | 2 | 3 | 3 | 2 | 9 | 5 | 8 | 22 |
| 128 | 28 | M | 2 | 2 | 1 | 4 | 1 | 2 | 4 | 2 | 5 | 5 | 7 | 11 | 23 |
