# Supplementary material for: The application of Item Response Theory on a teaching strategy profile questionnaire
Source: BMC Med Educ. 2010 Feb 10;10:14. doi: 10.1186/1472-6920-10-14 (PMC2830224; doi:10.1186/1472-6920-10-14)
Supplement: Additional file 2 — The parametric IRT approach. A description of the 2PL model and brief explanations of the interpretation of the parameters. [file 1472-6920-10-14-S2.DOC]

**Addional file 2**

*The parametric IRT approach:*

The basic concept of IRT rests upon the individual items rather than upon some aggregate of the item responses. In an IRT setting, the calculation is based on the probability that teacher s, s= 1,…, S, answers at level j, j=1,…,J on a J- point ordered scale, on item i, i=1,…,I.

#### *Location* = An item’s relative position or ‘difficulty’, d_i_ , on the underlying ‘activating scale’,

where i =1, 2, 3, for the items.

The frequency distributions of the responses indicate that certain items are ‘harder’ than others in the sense that it looks more difficult to endorse high levels. This finding is represented by the item difficulty. If two items show locations close to each other, this is an indication that they are both representing the same part of the underlying trait. The interpretation is that one of them is not necessary or that they confirm each other.

*Ability* = A teacher’s relative position, ‘ability’, on the underlying ‘activating scale’, i.e. the

relative degree of activating, or ability to activate. An *ability*, θ_s_, (degree of activating) is estimated for each teacher, s = 1,…,S. The term ability is commonly used to represent ‘individual measure’ in the IRT. In this study this term is replaced by ‘tendency’.

*Thresholds* = The category boundaries, as related to the item location, measured on the

underlying ‘activating scale’. c_k_ k=1, 2, 3, 4, is a common set of category boundary steps for all items within a dimension and related to the location by Σ c_k_ = 0. This is a reasonable restriction as the data set comprises just 59 subjects.

*Slope* or *Discrimination* = The slope of the item characteristic curve (ICC), which in essence means that the steeper the slope the better the power for an item to discriminate between low and high abilities in the neighbourhood of the item’s location on the activating scale. One measure of *discrimination*, a_i_ , is calculated for each item. If there are few items in the set and most of them show low discrimination, say considerably <1, it is an indication that the item set does not work in capturing the latent trait.

The specification of the so called Modified Rating Scale Model [5, pp 102-104]:

X ≥ k means ‘response in category k or above’, k=1,…,5.

For a teacher with *tendency* θ, the probability of response is modelled as

$$P(X\geq k,item i)=\frac{e^{a_{i}(\theta-(d_{i}-c_{k}))}}{1+e^{a_{i}(\theta-(di-c_{k}))}} where P(X\geq1)=1$$

Thus, the individually estimated *tendency* θ_s_ is, by means of the item parameters, directly connected to the probability of particular answers on the 5-point scale. The higher the θ_s_, the larger the probability of a high item score.

To further restrict the model to fewer parameters, the discrimination can be specified to be equal for all items (as is assumed in the sum score approach and the Rasch models, where a_i_ is set equal to 1).

The parameterization can be set up in alternative ways, but the one stated above is suitable for a type of questionnaires, such as in this study, where the answers constitute an ordered scale with equal number of levels for each item.

As no external reference is accessible, the actual data set is used as the reference set for construction of measures of the underlying dimensions.

*Tests of individual items and the item set.*

The test of an item is based on a comparison between observed frequencies and those predicted from the applied model. The constructed latent scale is divided in a set of intervals. The teachers are placed in the interval corresponding to their estimated tendency. For each interval, the frequencies of 1,2,3,4 and 5 are compared with what is predicted using the model. The differences between observed and expected frequencies are transformed into a chi-square statistic indicating the fit of the item. The item statistics are then summed up to form an item set chi-square statistic. For small samples this statistics are sensible to the choice of the number of intervals. With just 59 teachers, a small number has to be used, resulting in a not very efficient test statistic.

*Interpretation of the IRT parameter estimates.*

An analysis according to IRT transforms the items and their categories into item locations and related thresholds, estimated at the same latent scale as are the individual ‘teacher tendencies’.

AA and AM: In line with the low Mokken scalabilities, the large location SE:s indicate insufficient precision in identifying the teacher tendency. The large threshold intervals tell us that the item is not very efficient at the location but spread out over the tendency range. This usually means that even if the items are reasonable, they are not capable to estimate the teacher tendency with sufficient precision..

AR: In line with the negligible Mokken scalabilities, the low discrimination coefficients tell us that the analysis can not identify an unidimensional underlying trait representing the AR. This is also revealed by the large location SE:s, indicating that on the whole, there are difficulties in identifying a teacher tendency. The spread out thresholds is a consequence.

Figure 1 is an illustration of the positions of the AA items with their categories on the same latent scale as are the teachers. The thresholds in Figure 1 can be seen as the category thresholds between 1,2,3,4 and 5 in the original questionnaire.
